# Sustained Fixation Induced Changes in Phoria and Convergence Peak Velocity

**DOI:** 10.1371/journal.pone.0020883

**Published:** 2011-06-17

**Authors:** Eun H. Kim, Vincent R. Vicci, Sang J. Han, Tara L. Alvarez

**Affiliations:** 1 Department of Biomedical Engineering, New Jersey Institute of Technology, Newark, New Jersey, United States of America; 2 Department of Vision, Private Practice, Westfield, New Jersey, United States of America; 3 Department of Vision Rehabilitation, Kessler Institute, West Orange, New Jersey, United States of America; Smith-Kettlewell Eye Research Institute, United States of America

## Abstract

**Purpose:**

This study sought to investigate the influence of phoria adaptation on convergence peak velocity from responses located at different initial vergence positions.

**Methods:**

Symmetrical 4° convergence step responses and near dissociated phoria (measured at 40 cm from the subject's midline) were recorded from six subjects with normal binocular vision using an infrared limbus tracking system with a haploscope. Two different sustained fixations (1° and 16° convergent rotation along the subject's midline) were used to study whether phoria had an influence on the peak velocity of convergence responses located at two initial vergence positions (1° or ‘far’ steps and 12° or ‘near’ steps).

**Results:**

Phoria was significantly adapted after a sustained fixation task at near (16°) and far (1°) (p<0.002). A repeated measures ANOVA showed that convergence far steps were significantly faster than the near steps (p<0.03). When comparing convergence steps with the same initial vergence position, steps measured after near phoria adaptation were faster than responses after far adaptation (p<0.02). A regression analysis demonstrated that the change in phoria and the change in convergence peak velocity were significantly correlated for the far convergence steps (r = 0.97, p = 0.001). A weaker correlation was observed for the near convergence steps (r = 0.59, p = 0.20).

**Conclusion:**

As a result of sustained fixation, phoria was adapted and the peak velocity of the near and far convergence steps was modified. This study has clinical considerations since prisms, which evoke phoria adaptation, can be prescribed to help alleviate visual discomfort. Future investigations should include a systematic study of how prisms may influence convergence and divergence eye movements for those prescribed with prisms within their spectacles.

## Introduction

Vergence, a binocular eye movement, is responsible for attaining visual information located at different distances. The two primary inputs to the vergence system are retinal blur and disparity[1]. Retinal blur is the defocusing of images which stimulates the accommodative vergence system, whereas disparity is the difference between where a new target is projected onto the retina and the fovea. Disparity stimulates the disparity-vergence system and allows a person to perceive and maintain a single binocular vision. The eyes may rotate inward (convergence) or outward (divergence) to project the line of sight onto the same point of interest. Through the use of different instruments, one can study accommodative-vergence or disparity-vergence independently. A haploscope can systematically change where an object is projected onto the retina while maintaining a fixed focal length. Hence, when using a haploscope, any changes within eye movements can be assumed to be associated with the disparity-vergence system since the retinal blur stimulus is constant.

When a binocular stimulus is eliminated, such as when one eye is occluded while the other eye is fixating on a target located along midline, the occluded eye will decay to its dissociated phoria level. Near-dissociated phoria is quantified when the viewing or non-occluded eye is fixated on a target located at 40 cm from the subject's midline. Phoria can be classified as orthophoria, esophoria or exophoria, which are the absence, nasal or temporal rotation of the occluded eye, respectively. Phoria adaptation is a persistent modification in the eye alignment which can be due to a prolonged exposure to a binocular stimulus at different spatial depths.

A rich history exists in the literature that describes model-representation of the neural control of disparity-vergence [2], [3], [4], [5], [6], [7], [8], [9]. These models can be classified into two types: 1) negative feedback control or 2) preprogrammed control operating synergistically with a negative feedback control. The models with only negative feedback control are continuously modified such that the error between the input and output is adjusted until it is negligible. Negative feedback control models can be further separated into a single negative feedback control, [4], [5], [6], or into multiple channels where each channel is operated using separate negative feedback controls [3], [7].

One model that incorporates preprogrammed control is the Dual Mode Model [2], [8]. It is composed of a preprogrammed (transient) component and a feedback (sustained) component where the transient component influences the peak velocity of the response and the sustained component results in the accuracy of the final position [2], [8]. Most disparity-vergence models do not distinguish between convergence and divergence except for a positive or negative input stimulus. Hence, these models cannot predict any differences in convergence and divergence peak velocities. Previous studies have shown that divergence peak velocity is strongly dependent upon initial vergence position [10], [11], [12], [13], [14], [15], [16]. Depending upon where the responses are recorded, divergence peak velocity can be faster, slower or approximately the same as convergence peak velocity [11].

One model that does account for the differences between convergence and divergence movements was developed by Patel *et al.* using the Hodgkin-Huxley equation for membrane dynamics to predict vergence peak velocity [3], [10]. This model demonstrates that convergence and divergence responses have distinctive peak velocities at different ranges of spatial depth. Specifically, Patel's model predicts that with equal parameters for the convergence and divergence pathways, divergence responses at far would be slower (reduced peak velocity) than divergence responses at near while convergence responses at near would be slower than those at far. However, our current results and other studies suggest that the duration of time that a person is fixating on a target can influence the peak velocity in a disparity-vergence response [13], [17], [18]. Patel's model does not allow any of its parameters to change based upon how long a person is sustaining fixation on a prior target.

A few models have incorporated phoria adaptation in their design to account for modification of the disparity-vergence responses. Schor's model contains two portions, the fast-disparity vergence portion and the slow-disparity vergence portion [6], [19]. With sustained fixation, there is an increase in the output of the slow-disparity vergence portion that acts to change the phoria toward the current state of convergence angle; thereby reducing the load of the fast-disparity vergence portion [6], [19]. An alternate model proposed by Hung *et al.* employs a variable time-constant mechanism in which the neurons increase their time-constants proportionally to the duration of phoria adaptation [20]. Saladin's model has been also shown to account for direction during phoria adaptation by using a separate sensorimotor pathway for convergence and divergence [21]. However, these models do not accurately predict the influence of phoria adaptation on convergence and divergence peak velocity.

Studies have reported that divergence peak velocity is influenced by sustained fixation [13], [17], [22]. In one study, vergence responses were measured after 5, 30, 60 and 90 seconds of 6° sustained fixation where divergence peak velocity decreased significantly after 30 seconds or longer, compared to only 5 seconds of sustained fixation. Yet, the convergence peak velocity was unchanged for all the exposure durations up to 90 seconds [17]. Satgunam and colleagues (2009) studied the changes in 4° disparity-vergence step responses with an initial vergence position of 12° after five minutes of 12° (near) sustained fixation. They reported a decrease for divergence amplitude and peak velocity, and an increase for convergence amplitude and peak velocity, when comparing the data from before and after the near sustained fixation [18]. Satgunam and colleagues adapted for 5 minutes, (whereas Patel and colleagues adapted for up to 90 seconds) and demonstrated that when the sustained fixation was longer, it did influence the convergence peak velocity. However, neither study investigated the influence of the initial vergence position of the convergence step stimulus. Patel's neural network model predicted that convergence peak velocity would be faster when the initial vergence position was presented farther away from a subject's midline compared to an initial vergence position closer to the subject's midline [3], [10]. A systematic study to investigate whether the peak velocity of convergence is dependent on initial vergence position is warranted to test Patel and colleagues' model. In addition, using a sustained fixation position which is located at a different spatial depth compared to the initial vergence position of the convergence steps will allow us to study whether changes in phoria are correlated to changes in convergence peak velocity.

Satgunam and colleagues report that an esophoric shift in phoria as a result of near sustained fixation, leads to an increase in convergence dynamics [18]. They use a congruent stimulus; the position of the sustained fixation (direction of phoria shift) is similar to the initial vergence position of the 4° disparity-vergence steps. However, our study is designed to analyze the effects of both congruent and incongruent stimuli. For a congruent stimulus, the phoria is adapted to a similar visual location where the symmetrical vergence steps will be recorded. For incongruent stimuli, the position of the sustained fixation is mismatched with the initial vergence position of the 4° disparity-vergence steps. For example, the person's phoria is adapted at far and the convergence steps are recorded at near. Incongruent stimuli allow us to study greater changes in the phoria and convergence peak velocity from different initial vergence positions.

The purpose of our study is to test two different sustained fixation adapting positions (16° and 1°) and measure 4° convergence responses for two different initial vergence positions (1° and 12°). This experimental design allows us to investigate whether: 1) phoria influences convergence peak velocity, 2) convergence peak velocity is dependent on initial vergence position and 3) the change in phoria is correlated to the change in the peak velocity of convergence steps. In our study, change is defined as the phoria, or peak velocity, after near sustained fixation minus the phoria, or peak velocity, after far sustained fixation.

## Materials and Methods

### Subjects

The New Jersey Institute of Technology (NJIT) Institution Review Board (IRB) approved this study. All subjects signed written informed consent forms approved by the NJIT IRB in accordance with the Declaration of Helsinki. Six subjects, 22 to 65 years of age, who could easily perform the experiment described here participated in the study. All subjects had normal binocular vision defined as better than 50 seconds of arc by the Randot Stereopsis test and a near point of convergence (NPC) of less than 6 cm as described in our previous study [23]. Subjects were screened to ensure none of the subjects had anisometropia. Four subjects were emmetropes while two were myopes. Subjects S5 and S6 were myopic. Subject S5′s refraction was 2.75D and S6′s refraction was 2.25D for both the left and right eye. Both wore their refractive correction during the experiment. Our eldest subject (S1) had similar vergence peak velocities compared to the other younger subjects. Yang and colleagues also report no aging effects on the peak velocity of vergence [24]. Hence, subject S1′s data were included in the current study.

### Haploscope Setting and Experimental Setup

**Figure 1:**
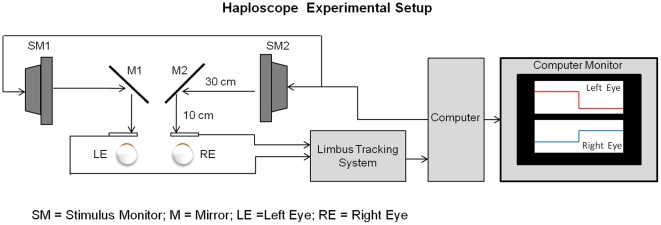
Haploscope experimental setup which stimulates retinal disparity while keeping accommodation constant. All visual stimuli were 40 cm away along midline for a constant 2.5D accommodative stimulus.

Visual stimuli were displayed using a haploscope. Two computer screens were used to generate a symmetrical disparity vergence stimulus, shown in figure 1. The stimulus was a green vertical line, 2 cm in height by 2 mm in width, and was presented on a black background. The two stimuli (green vertical lines) from each computer screen were projected onto the two partially reflecting mirrors and into the line of sight of the subject. Prior to the experiment, the stimuli from the computer screens were adjusted with the partially reflecting mirrors to calibrate the visual stimulus with real targets located at measured distances from the subject's midline. An inter-pupillary distance of 6 cm was assumed. During the experiment, only the visual stimulus displayed on the computer screens was seen by the subject. The subject's head was restrained using a custom chin rest to eliminate head movement and avoid any vestibular influences. No other visual stimuli were presented to the subject except for the visual stimuli presented on the computer screens. The stimuli screens were placed 40 cm away from the subject, hence the accommodation stimulus was held constant at 2.5D. A previous study showed that accommodative-vergence does not influence the initial peak velocity measurement [25]. Hence, vergence peak velocities observed in this present study will be assumed to be associated with the disparity-vergence system.

### Eye Tracking Instrumentation and Data Analysis

Eye movements were recorded using an infrared (λ = 950 nm) system manufactured by Skalar Iris (model 6500, Netherlands). All of the eye movements were within the linear range of the system (±25°). The left eye and right eye responses were recorded, calibrated and saved separately for offline data analysis. Eye movement digitization was preformed with a 12-bit digital acquisition (DAQ) hardware card (National Instruments 6024 E series, Austin, TX, USA). Visual stimuli and data digitization were controlled by a custom LabVIEW™ program (National Instrument, Austin, TX, USA). The eye movement signals were digitized at 200 Hz.

Calibration for the 4° disparity-vergence responses was composed of two points which were the initial and final vergence positions of the convergence step stimuli. The two-point calibration was viewed binocularly and was the initial and final combined vergence demand of the step stimuli. This calibration method was validated in our previous study [26]. The system has a high degree of linearity, within 3% between ±25 degrees horizontally [27]. Hence, a two-point calibration was adequate for this present study.

A custom MATLAB™ program (Waltham, MA, USA) was used for all data analysis. Left and right eye movement data were converted into degrees using the individual calibration data discussed above. Disparity-vergence was calculated by subtracting the right eye movement from the left eye movement. Any responses with blinks were excluded in the data analysis. Convergence peak velocity was computed using a two-point central difference algorithm [28]. Convergence responses are plotted as positive throughout the entire paper.

### Phoria Measurements

The subjects binocularly viewed a pair of vertical lines that stimulated 4.22° of rotation which corresponded to a target 40 cm away from the subject's midline, similar to the distance used clinically. The right eye was used to measure near dissociated phoria. A binocular target was presented for 2.5 seconds. Then, the right eye stimulus was extinguished and the eye position decayed to the steady state phoria level which was recorded for 15 seconds. This method of phoria measurement using our eye movement recording system was validated in our previous study [29].

A four-point calibration was utilized to assess the linearity of the eye movement recording system over the range of possible eye movement. A four-point calibration was used to ensure that the eye movement responses were within the calibration range since it was unknown prior to the study the extent to which each subject's phoria would be changed after the sustained fixation. The calibration points were observed monocularly with the right eye. The first calibration stimulus was 2° into the left visual field from the midline. The second calibration stimulus was on midline. The third and fourth points were 4° and 9° into the right visual field which equated to a potential phoria range of 3.5Δ esophoria to 15.8Δ exophoria. The right eye decay to phoria signal was converted into prism diopters because those are the units used clinically (One prism diopter  = 100tan (θ)).

### Experimental Protocol

**Figure 2:**
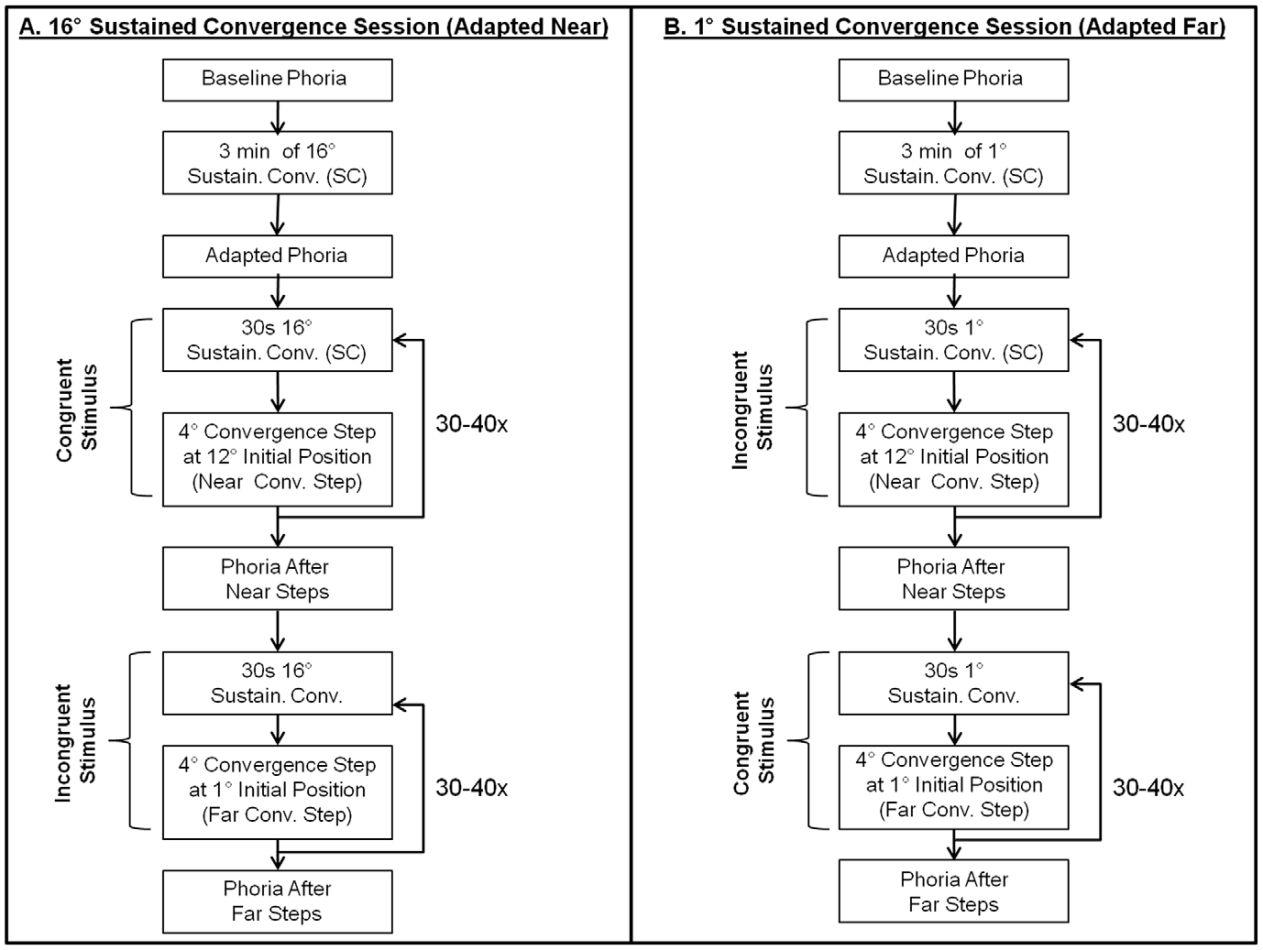
Experimental Protocol (see text).

During each session, baseline phoria was initially recorded. Baseline phoria was measured to determine whether the near or far sustained fixation adapted the phoria compared to baseline. The subjects fixated on a binocular target at 16° (near sustained fixation session, figure 2A) or 1° (far sustained fixation session, figure 2B) for three minutes. Subjects then performed a 4° convergence step starting at an initial vergence position of 12° and ending at 16° (near convergence step). The 30 seconds of sustained fixation after each convergence step was used to ensure that all the parameters of the vergence system that may have adapted after the initial three minutes of sustained fixation remained adapted during the collection of 4° convergence step responses. This was repeated 30 times. Subjects were asked to inform the experimenter if fatigue occurred. If the subject began to report fatigue, the experiment would end for that session. However, none of the six subjects reported fatigue. Phoria was recorded again after 30 near convergence steps to ensure that the phoria remained adapted; this is referred to as phoria after near steps throughout the paper. The subject then performed 4° convergence steps beginning at an initial vergence position of 1° and ending at 5° (far convergence step). Phoria was recorded again after 30 far convergence steps to ensure that phoria remained adapted; this is referred to as phoria after far steps throughout the paper.

Patel's model predicts that convergence peak velocity is faster for responses with a farther initial vergence position compared to responses closer to the subject or located near [3]. To ensure fatigue was not a confounding factor influencing convergence dynamics, near steps were recorded prior to the far steps. Fatigue in the form of repetitive eye movements has been reported to decrease peak velocity [30]. We presented far convergence steps in the latter part of the experiment because fatigue is more evident during the latter phase compared to the initial phase of the experiment. If fatigue had an effect in the experiment, it should decrease the convergence peak velocity. We recorded convergence at far during the latter part of the experiment because if the data support Patel's model, then the responses should be faster whereas fatigue would decrease the peak velocity.

Convergence steps were presented after a 0.5 second delay with an additional random delay of up to 1.5 seconds to avoid prediction. Predictive cues have been shown to increase the peak velocity of vergence eye movements [31], [32]. Responses were recorded for 3 seconds and data were saved for off-line analysis.

In summary, four types of 4° convergence step responses were recorded: 1) near convergence steps after a sustained fixation located at a 1° vergence position / far adaptation (incongruent stimulus), 2) near convergence steps after a sustained fixation position located at a 16° vergence position / near adaptation (congruent stimulus), 3) far convergence steps after a sustained fixation position located at a 16° vergence position / near adaptation (incongruent stimulus) and 4) far convergence steps after a sustained fixation position located at a 1° vergence position / far adaptation (congruent stimulus).

### Statistical Analysis

A linear regression analysis was used to assess the correlation between the change in phoria and the change in convergence peak velocity between the 1° and 16° sustained fixation tasks. The analysis was calculated using MATLAB^TM^. A paired *t*-test was used to determine whether the baseline phoria was significantly modified after the near and far sustained fixation tasks, and whether the baseline phoria was significantly different between the two days of experimentation. A repeated measures ANOVA was used to determine whether the peak velocity of the convergence steps was significantly different depending upon the initial vergence position of the convergence step (two initial vergence positions were investigated: 1° and 12°) and the sustained fixation task (near / 16° adaptation and far / 1° adaptation) using NSC2004 (Kaysville, UT, USA). Figures were generated using MATLAB™ and Excel software.

## Results

### Sustained Fixation Induced Phoria Adaptation

**Figure 3:**
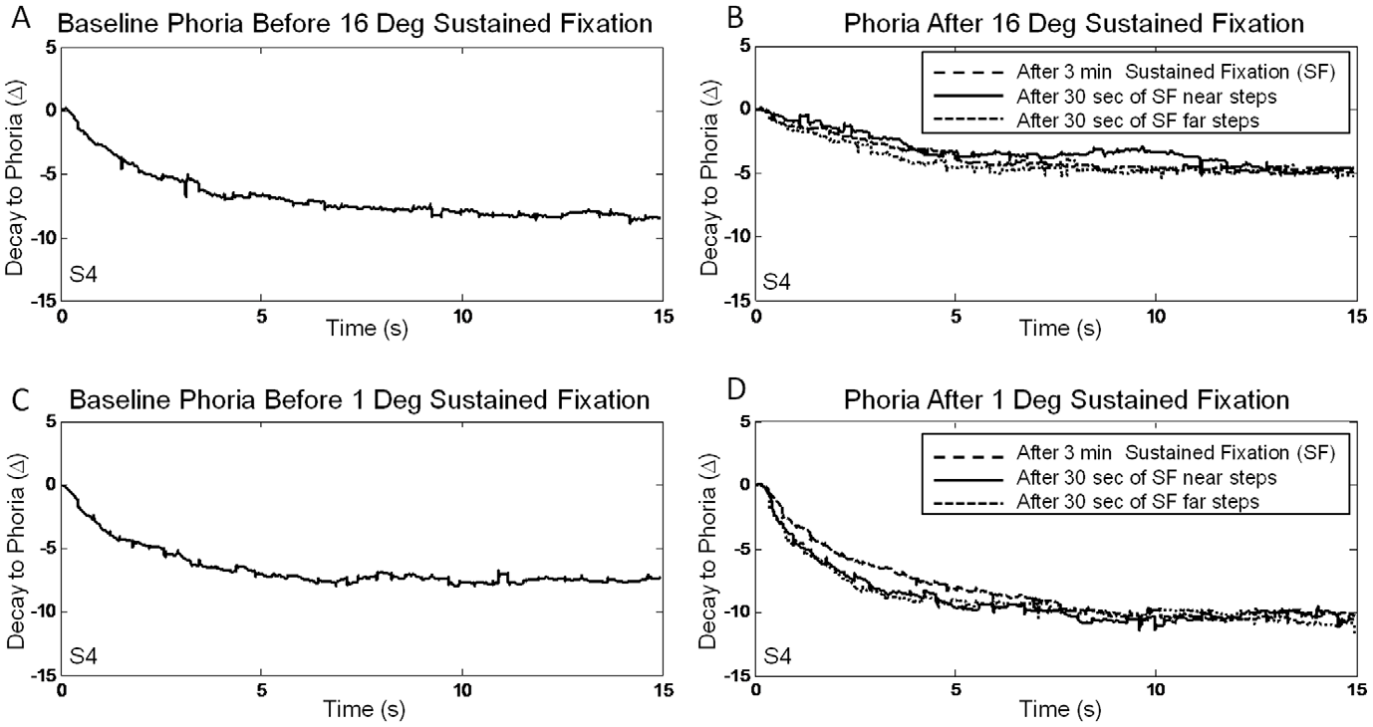
Right eye movement responses decaying to the phoria position during the baseline recording (left plots A and C). Adapted phoria responses are plotted on the right plots (plot B and D). Right eye movement responses decaying to the phoria position after 3 minutes of sustained fixation (long dashed line), after 30 seconds of sustained fixation during the near step phase (solid line) and after 30 seconds of sustained fixation during the far step phase (short dashed line) are shown in the right plots. The right eye movement response decaying to phoria is shown under two sustained fixation conditions, 16° or near adaptation (Plot B) and 1° or far adaptation (Plot D).

Typical right eye movement responses decaying to the subject's baseline phoria level, from S4 at the start of the experimental sessions, are shown in figure 3, plots A and C. These responses are typical single recordings. The right eye movement response decaying to the subject's phoria level was measured after three minutes of the sustained fixation task and is shown as a long dashed black line on the right plots. The right eye position response decaying to the phoria level was measured following the 30 convergence steps to determine whether phoria was still adapted. It is shown as a solid black line for the phoria response after the near convergence steps and as a dashed line for the phoria response after the far convergence steps.

**Figure 4:**
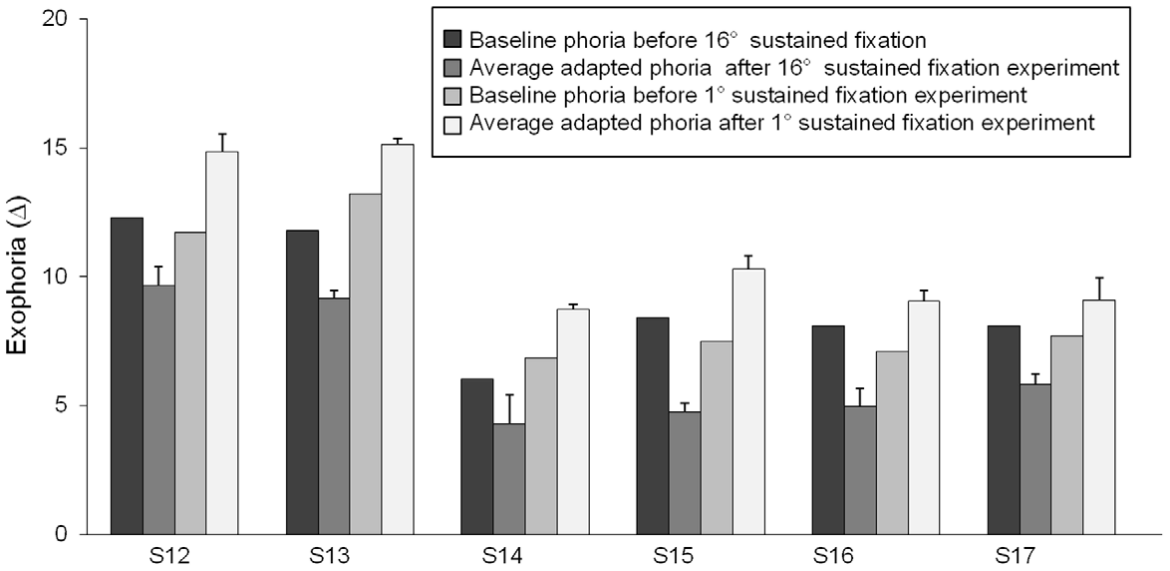
Summary per subject of baseline phoria before 16° sustained fixation (dark gray bar), baseline phoria before 1° sustained fixation experiment (light gray bar), adapted phoria after 16° sustained fixation task (medium gray bar) and adapted phoria after 1° sustained fixation task (white bar) for all six subjects. Adapted phoria measurements are the average of phoria measurements after the three minutes of sustained convergence, after near steps with an initial vergence position of 12° and after far steps with an initial vergence position of 1°. The error bars are one standard deviation from the mean.

The sustained fixation tasks altered the phoria level depending on the vergence position. A summary of the phoria measurements of all six subjects is shown in figure 4. All subjects became significantly more esophoric after the 16° sustained convergence task (p = 0.002). Similarly, after the far convergence task (1° vergence fixation), all six subjects became significantly more exophoric (p = 0.0008). The baseline phoria measurements were not significantly different between the two days of recording (p = 0.65).

### Effect of Sustained Fixation on Convergence Dynamics

**Figure 5:**
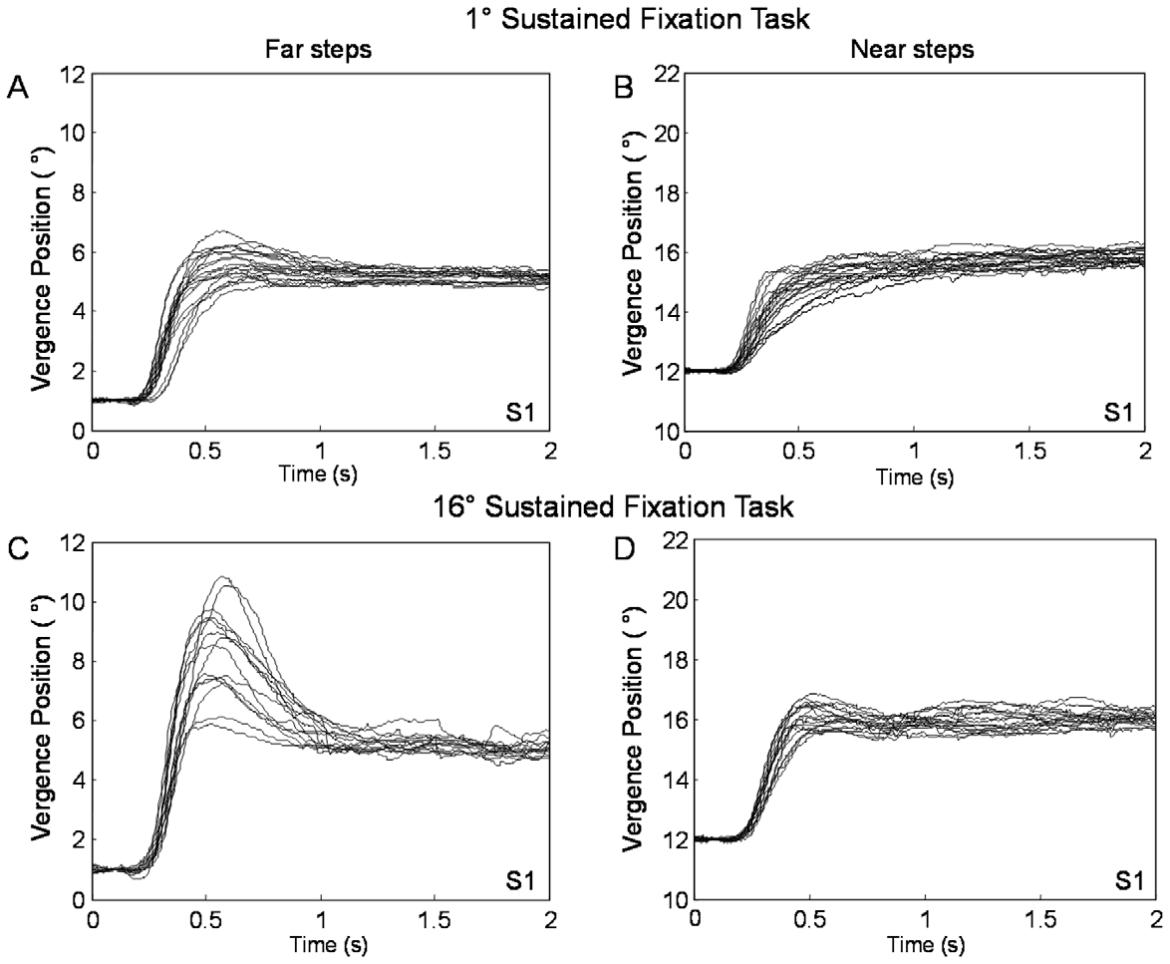
Typical ensemble convergence responses from a far initial vergence position of 1° (left) and from a near initial vergence position of 12° (right) after two sustained fixation conditions from subject S1. Plots A and B are after the 1° sustained fixation task / far adaptation; plots C and D are after the 16° sustained fixation task / near adaptation. The subject fixated on a sustained fixation target for 3 minutes and then each step response was recorded after 30 seconds of sustained fixation.

**Figure 6:**
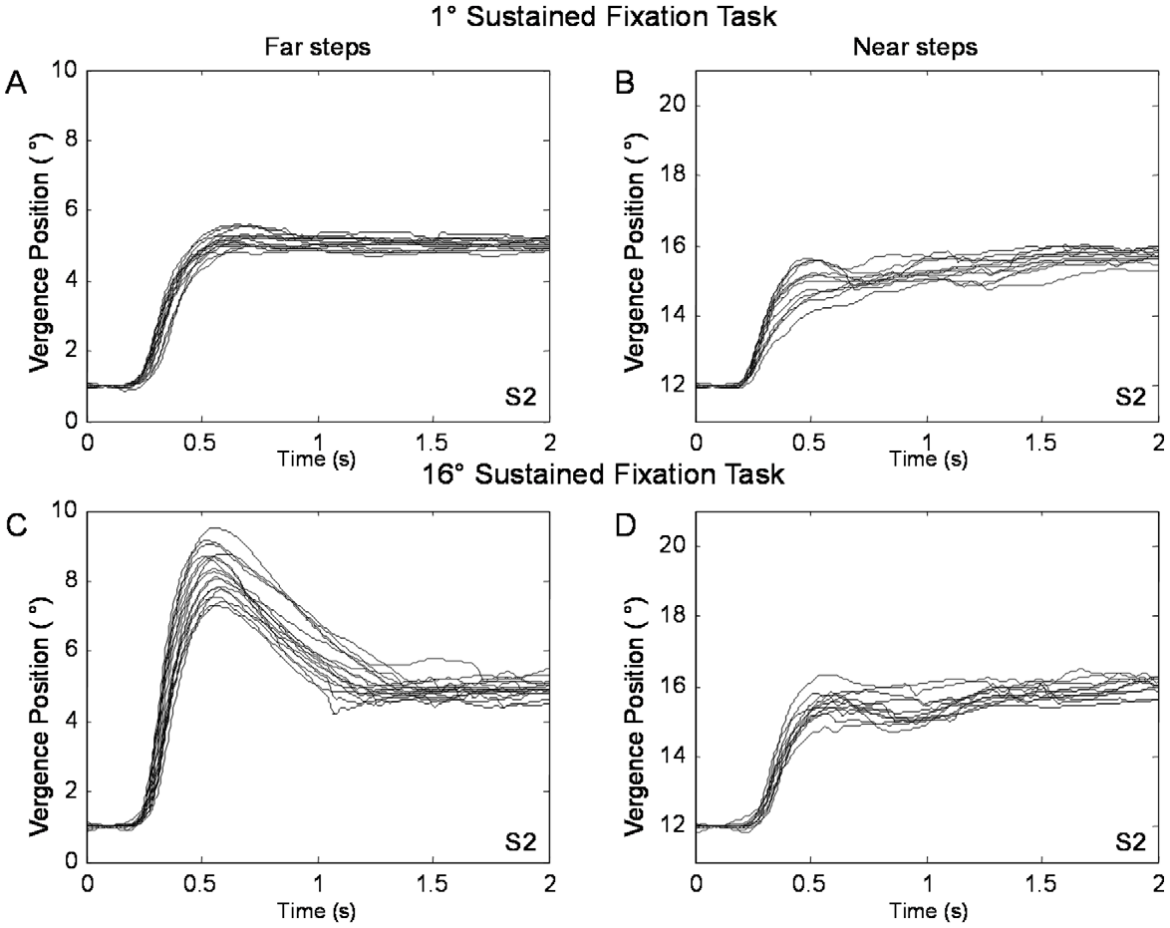
Typical ensemble convergence responses from a far initial vergence position of 1° (left) and from a near initial vergence position of 12° (right) after two sustained fixation conditions from subject S2. Plots A and B are after the 1° sustained fixation task; plots C and D are after the 16° sustained fixation task. The subject fixated on a sustained fixation target for 3 minutes and then each step response was recorded after 30 seconds of sustained fixation.

**Figure 7:**
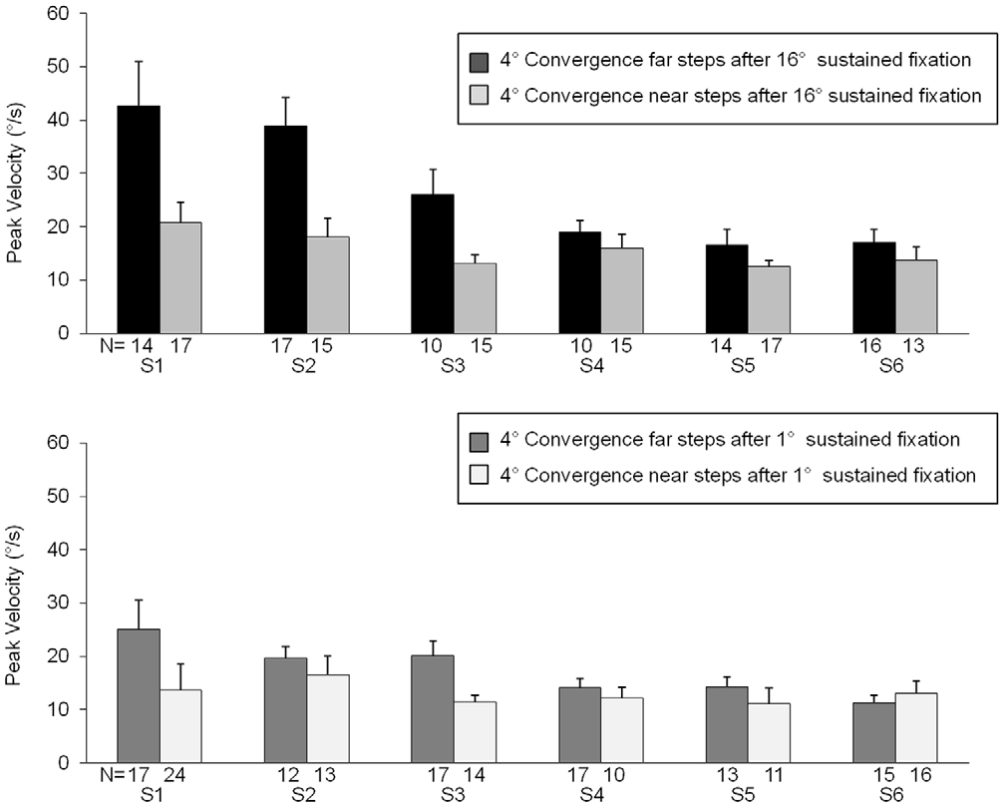
Summary of average peak velocities (°/s) with one standard deviation of all 4° convergence step responses after near 16° sustained fixation (top) and after far 1° sustained fixation (bottom). Far step data after sustained fixation of 16° (black), near step data after sustained fixation of 16° (light gray), far step data after sustained fixation of 1° (dark gray) and near step data after sustained fixation of 1° (white) are plotted. The number of samples is reported below the plotted data. When the phoria is maintained at one position, convergence responses with a 1° initial vergence position are faster than responses with a 12° initial vergence position.

Typical 4° convergence step responses from the four conditions (after sustained fixation of 16° or 1° vergence positions and convergence steps with an initial vergence position of 12° or 1°) of subjects 1 and 2 are shown in figures 5 and 6, respectively. The visual stimulus is the same, yet the dynamics of the responses varies depending on the initial vergence position and where the person was visually sustaining prior to the convergence steps (near phoria adapted compared to far phoria adapted). When the phoria was adapted to the same location, the peak convergence steps were dependent on the initial vergence position. For example, when the subject's phoria was near-adapted using the 16° sustained fixation task, the peak velocities of the far convergence steps were faster compared to the near convergence steps. Results are plotted in figure 7A. Similarly, when the subject's phoria was far-adapted using the 1° sustained fixation, the far convergence steps were faster compared to the near convergence steps as plotted in figure 7B.

A repeated measures ANOVA investigated whether the initial vergence position (near and far) and the sustained fixation adaptation (near and far) had a significant influence on the peak velocity of all 4° convergence steps. The peak velocities of the far steps were significantly greater than the peak velocities of the near steps [F (1, 5)  = 8.86, p<0.03]. Post-hoc analysis using the Bonferroni all-pairwise test indicated that the peak velocities of the convergence steps at near were significantly different than the peak velocities of convergence steps with a far initial vergence position. In addition, the average peak velocity of the steps after sustained near fixation was significantly faster than that of the steps after sustained far fixation [F (1,5)  = 11.84, p = 0.02]. These differences were confirmed with the post-hoc Bonferroni all-pairwise test. A paired *t*-test was used to compare the congruent stimuli. We compared the convergence peak velocity data between the two congruent stimuli (near steps after near adaptation and far steps after far adaptation). No statistical differences were observed (T = 1.0453, p = 0.3438) between convergence peak velocity at far after the far sustained fixation task and convergence peak velocity at near after the near sustained fixation task. The average convergence peak velocity with the corresponding standard deviation of near and far steps responses after the near and far sustained fixation tasks is summarized in figure 7. We did not observe significant differences between the latency of the responses for the different visual tasks.

### Correlation between Changes in Phoria and Convergence Peak Velocity

**Figure 8:**
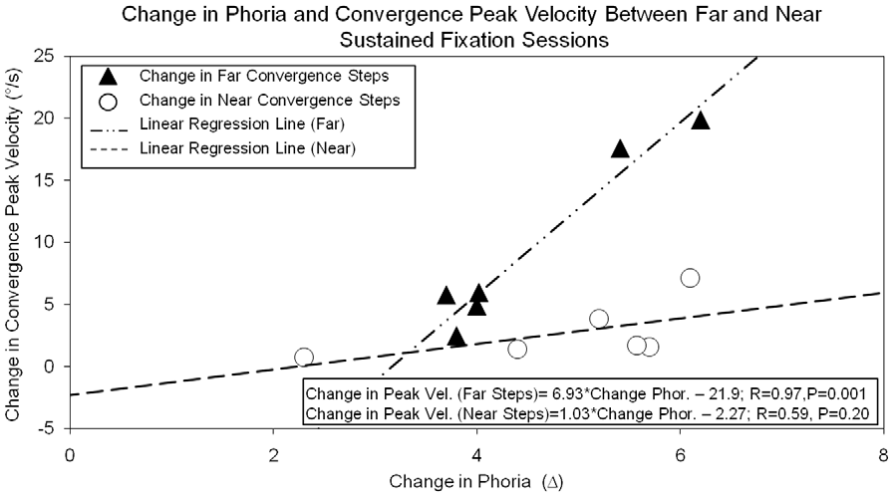
Linear regression plot of the change in convergence peak velocity (°/s) of the near steps (solid triangle) and of the far steps (open circles) as a function of the change in phoria level (Δ). The change in phoria level is defined as phoria after near [far] steps during the near sustained fixation task minus phoria after near [far] steps during the far sustained fixation task. Positive phoria changes refer to an esophoric shift in phoria. Similarly, the change in peak velocity is defined as the peak velocity of steps after the near sustained fixation task minus the peak velocity of steps after the far sustained task. Positive peak velocity changes refer to faster convergence peak velocity values during 16° (near) sustained fixation task compared to the 1° (far) sustained fixation task.

The correlation analysis between the change in phoria and the change in convergence peak velocity is plotted in figure 8. The baseline phoria level is not used for the correlation analysis. The change in phoria is described as the difference between phoria recorded after steps during the near sustained fixation task and the far sustained fixation task. This is shown in the experimental design in figure 2 where it is the data collected during the first experiment, or figure 2A, minus the data collected during the second experiment, or figure 2B. Hence, the change in peak convergence velocity for steps with a near initial vergence position is the difference in convergence peak velocity between steps after the 16° and the 1° sustained convergence task. The analysis compares the differences between the congruent responses (near steps with phoria adapted at near) to the incongruent responses (near steps with phoria adapted far). The analysis is repeated for far steps by comparing the incongruent responses (far steps with phoria adapted at near) to the congruent responses (far steps with phoria adapted far). Using the Pearson's correlation coefficient to quantify the correlation analysis demonstrates that the change in phoria is significantly correlated to the change in convergence peak velocity for far steps (R = 0.97, p = 0.001) where a weaker non-significant correlation is observed for the near steps (R = 0. 59, p = 0.20).

## Discussion

### 4° Convergence Step Responses are Dependent on Initial Vergence Position

The first purpose of the current study was to examine whether there were differences in convergence peak velocity at two initial vergence positions (1° and 12°). We observed that convergence peak velocities were faster at an initial vergence position of 1° (far steps) compared to an initial vergence position of 12° (near steps) recorded immediately after 16° (near) and 1° (far) sustained fixation tasks. Other studies have not reported initial vergence position dependence for convergence responses where the model from Patel and colleagues predicts convergence movements should be faster when located further from the subject [3].

Alvarez *et al*. (2005) studied the dependence of 4° disparity-vergence peak velocity on initial vergence position [11]. In this study, all subjects were presented with a variety of convergent and divergent stimuli. The initial vergence position of convergent and divergent stimuli ranged between 20° to 4°. They reported that divergence peak velocity was significantly dependent on the initial vergence position but convergence peak velocity did not significantly change. During this experiment, the stimuli were randomly intermixed. Hence, the phoria was not held relatively constant at one position. Similar findings were observed when studying converging and diverging ramp responses [12]. These experimental protocols may have obscured the convergence initial vergence position dependency predicted by Patel and colleagues' model [3]. The model proposed by Patel and his colleagues predicts that with equal parameters for the convergence and divergence pathways, divergence responses with a far initial vergence position would be slower than divergence responses with a near initial vergence position. Meanwhile, convergence responses with a near initial vergence position would be slower than responses located farther from the subject [3]. Patel and colleagues do report a divergence dependence on initial position but do not report significant changes for convergence when studying up to 90 seconds of sustained fixation [17].

When we compared congruent responses within this present study using a paired *t-*test, we did not observe any significant differences in peak velocity. However, by comparing incongruent responses with congruent responses, we do observe a convergence peak velocity dependence on initial vergence position. We speculate that by presenting the convergence stimulus at the same initial vergence position during a sustained fixation task repeatedly, we stimulated short term adaptive changes within the convergence system through phoria adaptation. This showed that convergence peak velocity was dependent on initial vergence position.

There are differences between the numbers of cells that respond to convergence compared to divergence movements and perhaps this may lead to the differences in the malleability of adaptation between convergence and divergence movements. There are distinct cells that encode for convergence and divergence within the midbrain where more convergence cells have been observed compared to divergence cells. These cells display a discrete burst of activity just before and during a convergence or divergence eye movement that is correlated with instantaneous vergence velocity [33], [34]. Since more convergence cells are observed within the midbrain compared to divergence, we speculate that differences in the cell population sizes may contribute to the differences in the ability to change convergence peak velocity compared to divergence movements.

The visual system does encode for relative disparity where the brain processes information about relative position of a target with respect to the person. Specifically, there is evidence from the single cell recordings in primates of disparity tuned cells that modulate their activities for near and far positions within the primary visual cortex [35], [36], [37]. Perhaps the neural activity encoding of near, far and ‘zero disparity’ stimuli observed within primate studies [13] may in part be the stimulus that drives the differences in average disparity-vergence peak velocity that have been demonstrated in this present study for convergence as well as previous research investigating divergence [10], [11], [12], [13], [14], [15].

### Modification of the Transient Convergence Due to Sustained Fixation and Subject Variability

Another goal of this research was to study whether convergence peak velocity was modified due to phoria adaptation stimulated through sustained fixation. We observed that subjects demonstrated either hypermetric or hypometric movement in the transient portion of convergence responses depending upon the type of the incongruent stimulus. During the 16° sustained fixation task with a far convergence step, hypermetric responses were present in the transient portion. During the 1° sustained fixation task with a near convergence step, hypometric responses were present during the transient portion. This study supports that when the immediately preceding sustained fixation is mismatched to the initial vergence position of the convergence step, the transient portion of the convergence response is modified compared to responses when the sustained fixation is similar to the initial vergence position of the convergence step.

Another similar study to ours by Satgunam *et al.* reported that an esophoric shift in phoria increases both the amplitude and peak velocity of convergence step responses [18]. Our current study extends Satgunam and colleagues' research by showing that an esophoric shift in phoria (due to 16° sustained task) results in an increase in convergence peak velocity at both near (12°) and far (1°) initial vergence positions. We also observed that an exophoric shift in phoria (due to 1° sustained task) resulted in a decrease in convergence peak velocity at both near and far initial vergence positions.

Interestingly, the change in convergence dynamics was more apparent in some subjects compared to others. For example, S1 and S2 who were both emmetropes at this viewing distance showed greater change in the transient portion of convergence responses while other subjects exhibited a similar behavior but to a lesser extent. The variability in convergence dynamics among the six subjects suggests that some subjects may possess an innately faster vergence system compared to others. While different factors may attribute to the subject variability in vergence dynamics, we speculate that phoria may be a factor, explaining in part why some subjects have greater changes in convergence dynamics compared to other subjects. In addition, we do not feel that age was a contributing factor because we redid our analysis with only the five younger subjects and the results do not substantially change.

### Correlation between Change in Phoria and Convergence Dynamics

To better assess the relationship between phoria and convergence dynamics, a correlation analysis was conducted between the change in phoria and the change in convergence peak velocity. The result demonstrated that the change in convergence peak velocity was highly correlated to the change in phoria for far steps but was moderately correlated for near steps. The near steps were collected at the beginning of the experiment so it is unlikely that fatigue was a confounding variable that led to a reduction in correlation for the near steps compared to the far steps analysis. However, the results support that the change in phoria was moderately correlated for near steps.

Furthermore, our analysis suggests that subjects who showed a greater change in phoria also showed a greater change in convergence peak velocity. Single cell primate studies have researched phoria and disparity vergence eye movements. Cells located within the vicinity of the midbrain have been identified to modulate their activity based upon phoria adaptation [38] as well as on convergence velocity [39]. Lesions to the oculomotor vermis in primates have resulted in a decrease in vergence velocity and a reduction in phoria adaptation [40], [41]. A direct link between the dorsal vermal outputs of the cerebellum to the midbrain via the caudal fastigial nucleus has been reported, suggesting structural connectivity between the two regions [42]. In addition, human case studies report reduced vergence dynamics in patients with cerebellar lesions, particularly those within the vermis [43]. The signal that drives and controls the modification in vergence dynamics as well as phoria needs further study. Likewise, the rationale of why some subjects have a greater change in phoria leading to an increased change in convergence dynamics requires additional investigation.

### Clinical Implications of Phoria Adaptation and Vergence Eye Movements

A conventional treatment for binocular dysfunctions such as convergence insufficiency (CI) is using a prism correction within a spectacle which stimulates a phoria / prism adaptation; however a common clinical observation for CI patients is that the prism reduces the patient's symptoms initially, but it does not have sustained effects [44]. In addition, Brautaset and Jennings report phoria adaptation is reduced in CI patients where they suggest CI patients may have a generally reduced horizontal phoria adaptation mechanism [45]. However, these patients demonstrate improvements in their ability to perform phoria adaptation [46], [47] and increases in convergence peak velocity after oculomotor training [23], [48]. Future studies are needed to understand how oculomotor training improves the ability of both the disparity-vergence and the phoria systems to adapt in binocular dysfunctions such as convergence insufficiency.

### Future Direction of Disparity-Vergence Models

Some disparity-vergence models do include components that can adjust and suggest that adaptation occurs via a recruitment mechanism or a time-constant modulation [8], [19]. However, these models do not incorporate any mechanism by which vergence peak velocity and phoria can adapt together as a result of sustained fixation. Therefore, a model is needed to describe convergence and divergence independently and account for the influence of sustained fixation on disparity-vergence peak velocity. One possibility is to modify the transient / preprogrammed (open loop) component of the Dual Mode Model [8] by altering the width and / or height of the transient component dependent upon changes in phoria and on the initial vergence position of the vergence step. This current study supports that a new model-representation of the disparity-vergence system, one that incorporates phoria in addition to the direction and initial vergence position of the stimulus, is needed to account for changes in disparity-vergence peak velocity observed within our experimental data.

### Differences in Baseline Phoria Measurements

Lastly, although we did not observe a significant difference, the baseline phoria measurements were not identical on each day. Lee *et al*. also observed differences in phoria when data were recorded on different days [13]. Phoria can be influenced by many factors, such as near work [49]. Hence, the changes in baseline phoria could potentially be due to the amount of near work the subject was performing prior to the experiment. Other potential factors that may lead to variability of the baseline phoria include physiological changes, such as the amount or degree of fatigue, inattention, cognitive demand and / or accommodation from different days [22], [50].

### Conclusion

In summary, phoria was altered depending upon the far and near adapting sustained fixation positions. Convergence peak velocity showed a dependency on the initial vergence position where responses farther from the subject (far steps) were faster than responses closer to the subject (near steps). Phoria adaptation, as a result of sustained fixation, modified convergence peak velocities. Peak velocity from convergence steps measured after near phoria adaptation was faster than responses after far phoria adaptation. The linear regression analysis supports that the change in phoria and the change in convergence peak velocity for the far steps were significantly correlated.
